# How Stakeholder Pressure Affects the Effectiveness of International-Local Nongovernmental Organization Collaboration in Localization of Humanitarian Aid

**DOI:** 10.1177/08997640231196886

**Published:** 2023-09-26

**Authors:** Mohammad Moshtari, Ghasem Zaefarian, Evelyne Vanpouke

**Affiliations:** 1 Faculty of Management and Business, Tampere University, Finland; 2 Leeds University Business School, University of Leeds,UK; 3 Solvay Brussels School of Economics and Management, Université libre de Bruxelles, Belgium

**Keywords:** nonprofit organizations, humanitarian organizations, humanitarian operations, collaboration, aid localization, capacity development, stakeholder theory, survey

## Abstract

Collaborative engagement between international and local nongovernmental organizations (NGOs) has recently been promoted as an effective strategy to enhance internal process strengths but less as a strategy to localize humanitarian aid programs; a grand strategy that aims to strengthen local capacity, develop local capabilities, and boost regional humanitarian project performance. While stakeholders deem to play an important role in leveraging the efficiencies of such collaborative engagements between international and local actors, there is limited empirical knowledge about how stakeholder pressure affects the association between the collaboration–performance association within international and local NGOs. Drawing on stakeholder theory, we propose a model to examine the role of donors, media, and governments, three major stakeholders noteworthy because of their power and legitimacy to moderate the collaboration–performance association in this NGO context. We test our hypotheses across a series of samples collected at both international and local NGOs in 2015 and 2020. From a practical perspective, we discuss how the traditional role of NGOs as implementers of aid programs is shifting toward a new role as conveners and capability builders.

## Introduction

Proficient humanitarian aid projects require intensive collaboration among international and local humanitarian organizations. More specifically, collaborative engagement between international nongovernmental organizations (INGOs) and local nongovernmental organizations (LNGOs) not only increase the internal process strengths of the project such as reducing the overall service costs or increasing quality (Fawcett et al., 2012) but could also increase the inclusion and empowerment of national and local actors, enabling the localization of aid programs. This localization process involves INGOs empowering instead of commanding LNGOs and can vary in its degree between strong localization (when LNGOs lead and INGOs assist), medium localization (when LNGOs and INGOs are equal partners), and weak localization (when LNGOs act as agents dictated by INGOs) (Tran & AbouAssi, 2020). Moreover, while INGOs traditionally designed and implemented humanitarian aid projects via own channels, mainly focusing on process efficiency, there has recently been a major strategy shift toward creating local capacities. Strong localization should create projects in which INGOs play a supporting coach or mentor role that reinforces rather than replaces LNGO capacities (Featherstone, 2016). Opening the humanitarian system through this localization process is particularly useful because it increases the value of the relief and development projects toward local beneficiaries in terms of project performance, as well as creating local capabilities (Agenda for Humanity, 2016; Schech et al., 2020). More specifically, it could enable stronger ties and engagements from local communities (beneficiaries or local aid organizations) and better knowledge transfers toward these local communities. This process of localization is particularly important as currently 60% of all international donations end up at INGOs, which frequently channel it through different collaborative schemes to local communities (Development Initiatives, 2020), often via their local INGO offices, but more recently, also more frequently via LNGOs. The Charter for Change even committed to providing 25% of the INGO donations via these LNGO collaborations (Agenda for Humanity, 2016).

The need for fostering INGO–LNGO collaborations has not only been recognized by humanitarian organizations but also by humanitarian stakeholders, such as donors, governments, and media. Explicit stakeholder support for these collaborative engagements between INGOs and LNGOs could impact the strength of this localization process, eventually impacting the effectiveness of the humanitarian aid projects. To understand this relationship between collaborative engagement and either strategic outcomes or improved local capacity, it is important to analyze this relationship within the context of external stakeholders. More specifically, stakeholder theory suggests that organizations are influenced by different stakeholders, a pressure that can affect managers’ plans, actions, and decisions (Freeman, 1984). In a humanitarian context, stakeholders, such as donors, media, and governments, may influence the effectiveness of collaborative engagements, and consequently the strength of the localization of aid, between international and local actors. These three stakeholder groups, that is, donors, media and governments, are specifically noteworthy in this NGO context as they are able to pressure action for more collaborative engagement as a result of their power, legitimacy, and urgency (Mitchel et al., 1997). More specifically, INGOs might pay attention to these three stakeholder pressures, as doing so enables them to gain legitimacy, collect future donations, mobilize resources, or gain access to affected areas. Also, LNGOs might pay attention to these stakeholders’ requests for more collaborative engagements with INGOs because doing so may influence their credibility in local communities, impacting future donations and survival. While previous research indicated that stakeholders may affect the success of INGO–LNGO collaborations (Adem et al., 2018), it has not considered how this collaboration–performance link is impacted by stakeholders’ direction, strength, or impact on both INGOs’ and LNGOs’ performance; in other words, how donors, media, and governments moderate the relationship between collaborative engagement and strategic outcomes/improved local capacity.

Against this backdrop, our research aims to investigate the efficacy of stakeholder pressure by analyzing how perceived stakeholder pressure for INGO–LNGO collaboration differentially moderates the path from collaborative engagement to both humanitarian project performance and local capability enhancement for INGOs and LNGOs. This moderation effect might be present as these stakeholder pressures might encourage NGOs to stay in the relationships long enough for them to be successful. To address this issue, we adopt a theory-testing approach using survey data from eight different countries in 2015. In Study 2, we repeat the analysis with new samples in 2020 to investigate whether the results of both studies are consistent with each other. Our surveys capture the viewpoints of INGOs and LNGOs separately in both studies.

## Theoretical Background and Hypotheses

### Collaborative Engagement and Its Performance Outcomes in a Humanitarian Setting

Interorganizational collaboration is an interdisciplinary area that has been studied in several branches of management research. Prior studies suggest that collaborations are positively associated with firm performance (Fawcett et al., 2012; Leuschner et al., 2013). Similarly, in the public and nonprofit sectors, Gazley (2010, p. 53) suggested that collaboration can yield many benefits, including “economic efficiencies, greater service quality, organizational learning, access to new skills, diffusion of risk, improved public accountability, the ability to buffer external uncertainties, and conflict avoidance.”

The increase in collaborations between INGOs and LNGOs mainly concerns local operational aid delivery. While these collaborations can count on LNGOs as implementation partners (Altahir, 2013), INGOs also benefit from local organizations’ contributions to other tasks within humanitarian supply chains, such as needs assessment, context and capacity analysis, program design fundraising, and procurement (De Geoffroy et al., 2017). Local organizations have easier access to local information, such as affected populations and their needs, as well as the available local resources, enabling them to better identify and employ local service providers. In addition, local organizations know the context and the culture and are experienced in working with local communities (Unerman & O’Dwyer, 2010).

Increasingly, INGOs acknowledge local actors’ complementary roles, creating possibilities for INGO–LNGO humanitarian collaborations to rebuild national capabilities, increase the affected communities’ resilience, and over the long term, provide sustainable humanitarian assistance (Street, 2011). While it is important to establish a long-term partnership and to create these process improvements, it is equally important to create a sustainable outcome based on building capabilities at the level of the LNGO and not dependency on the international community (Altahir, 2013).

In this research, we use the concept of collaborative engagement (Zacharia et al., 2011), which refers to the strength of collaboration activities, operationalized by assessing the level of joint planning (e.g., forecasting, product design, fundraising campaigns), collaborative communication, joint decision-making, resource sharing, and joint project implementation. Through collaborative engagement, NGOs become involved in joint planning (e.g., forecasting, needs assessment, and context analysis) to gain more information about their counterparts and their needs (Villena et al., 2011). They also inform one another about any issues or events that may affect their operational plans. In addition, partners can exchange valuable or complementary resources, such as best practices, knowledge, and infrastructure. The autonomy and equity of NGOs are respected, and open or two-way collaboration is exercised through joint activities. Consequently, a project or program conducted within a collaboration reaches higher levels of quality, with lower costs and shorter delivery and lead times.

To carry out a more comprehensive assessment of these collaborative outcomes, we propose extending the scope of these potential benefits beyond operational aspects by including strategic outcomes resulting from collaborative engagement, such as improvements in local partner capabilities. In addition to successfully implementing humanitarian projects that deliver aid to beneficiaries, INGOs try to empower LNGOs to carry out the most essential tasks of future operations independently (Charter for Change, 2016). Acquiring these project-development and implementation skills can contribute to the autonomy and equality of LNGOs or might even empower them to apply for funds directly from donors, enabling them to evolve into viable, independent participants in the aid sector (Alcacer & Oxley, 2014). In addition, knowledge transfer might increase the efficiency and innovation related to different operational activities and improves the quality of outsourced activities. For instance, BRAC and Proshika were local partners of Swedish NGOs in the period after Bangladeshi independence, but they later developed into independent organizations that are among the largest and most successful NGOs in the world (BRAC, 2016; Lewis, 1998).

### Stakeholder Pressure

To better understand the relationships between NGOs, as well as between NGOs and society, stakeholder theory has become increasingly important to many scholars (Freeman, 1984). A stakeholder is “any group or individual who can affect, or is affected by, the achievement of the organization’s purpose” (Freeman, 1984, p. vi). Indeed, every stakeholder has different expectations of the NGO and demands different returns, such as money, sustainability, ethics, or quality. A stakeholder is an individual or group with one or more stakes in a humanitarian NGO. Previous research showed that donors, governments, and media have a huge influence on a humanitarian NGO’s operations (Schiffling & Piecyk, 2014). These secondary stakeholders have been chosen due to playing a vital role in raising awareness and donations, as well as sometimes hindering operations (Van Wassenhove, 2006). Moreover, while these external stakeholders have an indirect or derivative connection to the NGO, the NGO may have limited or no formal responsibility toward them. Indeed, these external stakeholders, that is, governments, donors, and media, could have significant influence on INGO–LNGO collaborations, particularly in relation to an organization’s reputation and public status.

It is important to note that the pressure of a stakeholder, in terms of power, legitimacy, and urgency, can change with circumstances, as well as the issue at hand (Schiffling & Piecyk, 2014). Indeed, external stakeholders may quickly increase their pressure on the NGO. This happens, for example, when the media calls for a demonstration, boycott, or other action, leading to a crisis for the NGO, such that the NGO must respond immediately to their criticisms. In addition, the media today has a great deal of power and the ability to easily transform a secondary stakeholder into a primary stakeholder in a matter of hours. Also, governments can pressure NGOs because of their power as donor or through legitimacy as the official requesting body for assistance (Kovacs & Spens, 2007). Finally, donors are very salient stakeholders as they have the power to provide funding and to create urgency by imposing deadlines (Sandwell, 2011). Therefore, it is crucial for an organization to know its stakeholders and their priorities. A stakeholder that is perceived by the NGO as possessing more of these characteristics exercises a higher pressure on the NGO. Because NGOs depend on multiple external stakeholders, such as donors, governments, and the media, they can have direct or indirect impacts on the NGOs’ effectiveness in collaborating.

### Hypothesis Development

#### Donor Pressure and INGO–LNGO Collaboration

In contrast to the commercial sector, humanitarian NGOs typically do not receive any revenue from beneficiaries for the delivery of their services. NGOs often depend on donations to cover all or nearly all of their organizational and operational costs. Accordingly, donors are powerful because they influence the use of resources or withhold resources to force NGOs to adopt specific practices (Freeman, 1984). Two mechanisms explain how donors affect the link between INGO–LNGO collaboration and performance. A first mechanism can be explained by NGOs’ eagerness to prove to donors that they are cost-effective and working hard to build local capacity by increasing their effectiveness via local collaborations (United States Agency for International Development, 2012). Because of the limited resources that donors can provide to affected regions, they often force NGOs to focus on enhancing the efficiency and impact of their humanitarian operations. As such, donor pressure to collaborate locally is usually the driving force for improving the efficiency of INGOs’ collaborations with LNGOs in humanitarian operations. Today, most donors increasingly want to be informed about how donations are used on the ground, as well as the local sustainability of these projects. Moreover, donors increasingly rely on past performance in their future resource allocations. When donor pressure for international–local humanitarian NGO collaboration is perceived to be high, NGOs understand that their performance is closely monitored and that future donated resources depend highly on how they perform. In addition to the pressure for future donations, NGOs also want to be recognized by donors and showcase their achievements. To do this, they might feel pressured to show that collaborations between INGOs and LNGOs are effective and are creating benefits for the local community. As such, to legitimize their actions, NGOs work hard to enhance the productivity of their collaborative actions by showing their efficiency and sustainability (Charter for Change, 2016).

Because INGOs are the main contact point and receivers of donations, they might feel strong pressure from donors to collaborate with LNGOs. As such, INGOs might be willing to put extra effort into collaborating with LNGOs, which enables them to communicate superior humanitarian outcomes in terms of both project performance and local partner capabilities, as well as securing future donations. However, LNGOs also feel this pressure from donors: Via the INGOs, they indirectly depend on donors to receive funding. In periods of reduced funding possibilities, these LNGOs are more receptive to fulfilling these requirements from donors. As such, we can formulate the following hypotheses:

**Hypothesis 1 (H1):** Donor pressure strengthens the positive association between collaborative engagement among INGOs and LNGOs and humanitarian performance of the INGOs in terms of both project performance and local partner capabilities.
**Hypothesis 2 (H2):** Donor pressure strengthens the positive association between collaborative engagement among INGOs and LNGOs and humanitarian performance of the LNGOs in terms of both project performance and local partner capabilities.

#### Government Pressure and INGO–LNGO Collaboration

Another group of stakeholders is the host government, whether national or local. Although host governments and authorities do not directly control NGO resources, they have a significant amount of power to give—or withhold—permission for organizations to enter an area and dictate how to operate in it. Governments can affect the access of NGOs to the field and eventually increase or impede the timeliness of their response (ALNAP, 2016). Some governments may facilitate the collaborative operations of INGOs with LNGOs, but others may enforce regulations for reasons of control (political motives) or regulate specific operations, such as health care or medical supplies (Dube et al., 2016). They can also restrict the collaborative operations of international–local NGOs with a history of distrust or limit the collaborations of non-professional or novice NGOs (ALNAP, 2016).

Host governments often have the power to establish regulations to encourage INGOs to improve their collaboration with LNGOs. These pressures can force NGOs to further engage and follow up joint achievements through additional information sharing, effective communication, and more intensive cooperative planning. Eventually, these pressures can leverage or impede the ability of international and local partners to successfully implement a project and increase local partner capabilities. For example, INGOs have reported that they must show joint collaboration with LNGOs to guarantee their eligibility to work in Jordan (Adem et al., 2018). To further stimulate this, host governments can establish regulatory frameworks to make it easier for international actors to collaborate with local ones. Government authorities may monitor an INGO’s use of existing local infrastructure and supplies, along with its training practices, with the goals of raising LNGOs’ skills, protecting local industries, and transferring expertise to local communities. Kunz and Reiner (2016) argued that close collaboration with a well-regarded local organization for in-country operations can help address the host government’s possible political or operational concerns of an INGO. However, when government pressure is low or negligible, the collaborative engagement of INGOs with LNGOs could be less effective, as INGOs may be less motivated to engage in knowledge and skill transfer with LNGOs. Because local governments often protect LNGOs and their local capabilities, governments might use their regulatory power to affect the activities of INGOs in favor of these LNGOs, creating pressure on local–international humanitarian NGO collaboration. Therefore, we hypothesize the following:

**Hypothesis 3 (H3):** Government pressure strengthens the positive association between collaborative engagement among INGOs and LNGOs and humanitarian performance of the INGOs in terms of both project performance and local partner capabilities.
**Hypothesis 4 (H4):** Government pressure strengthens the positive association between collaborative engagement among INGOs and LNGOs and humanitarian performance of the LNGOs in terms of both project performance and local partner capabilities.

#### Media Pressure and INGO–LNGO Collaboration

The media are often perceived as important stakeholders for humanitarian NGOs because they can act in favor of or against an NGO’s practices, strengthening the impact of these practices, such as INGO–LNGO collaborations. At the same time, the increasing use of social media may also strengthen the impact of media on perceptions concerning humanitarian organizations and collaborations among them. The media can pressure NGOs to collaborate—mainly in terms of urgency—by influencing public opinion about their actions, which eventually influences NGOs’ relations with key stakeholders, such as donors or host governments. For example, Eftekhar et al. (2017) showed that humanitarian organizations are sometimes reluctant to coordinate horizontally among peers because this might dilute media attention that individual humanitarian organizations might receive, and consequently, decreases future donations. However, in terms of vertical coordination, such as for INGO–LNGO collaborations, improved coordination may increase future donations because it could result in more positive media attention for both partners, and eventually, more donation opportunities. More specifically, in an international context, the media often brings the news directly from the local beneficiaries to show what is happening on the ground. This pushes INGOs and LNGOs to better collaborate, and eventually, a better flow of donations toward the beneficiary emerges; this may create better media exposure toward donors for the INGO, as well as a positive attitude on the part of the INGO to extend the agreement with the LNGO. The opposite might also be true. Because a lack of effective and close collaboration in the humanitarian sector can lead to delays in providing services, cause ineffective aid distribution, or even result in beneficiary injury or death, it can eventually lead to negative media attention. To avoid such unwelcome developments, NGOs expend substantial effort on making their collaborations between international and LNGOs successful, which will improve the perception of local stakeholders and NGOs’ overall public image.

Local communities and beneficiaries are the recipients of humanitarian services. Although they often do not pay for these services, their satisfaction and complaints are frequently reported by media. For example, beneficiaries now have access to a variety of social networks to gather information about NGOs’ services and performance and convey their opinions to governments and donors via the media. Given their importance in providing visibility, the media can pressure NGOs, and INGOs even more so, to influence the urgency of collaboration between humanitarian partners and the decisions made about NGO practices (Gunningham et al., 2004). More specifically, pressure from the media shifts more power toward the LNGOs, resulting in a more balanced INGO–LNGO collaboration. Thus, the following hypotheses are formulated:

**Hypothesis 5 (H5):** Media pressure enhances the positive association between collaborative engagement among INGOs and LNGOs and the humanitarian performance of the INGOs in terms of both project performance and local partner capabilities.
**Hypothesis 6 (H6):** Media pressure enhances the positive association between collaborative engagement among INGOs and LNGOs and the humanitarian performance of the LNGOs in terms of both project performance and local partner capabilities.

## Study 1 and Data Analysis

### Data Collection

The empirical context of our research is NGOs involved in humanitarian operations. The website of the Office for the Coordination of Humanitarian Affairs (OCHA) provided contact information for NGOs participating in eight country-based clusters around the world (i.e., Afghanistan, Congo, Haiti, Indonesia, Kenya, Niger, Sudan, and Sri Lanka). This resulted in a sample frame of 843 international and 927 LNGOs active in humanitarian aid services.

In the survey introduction, we informed respondents that “a collaborative relationship refers to a partnership process where one international and one local humanitarian NGO share resources (e.g., information, expertise, and infrastructure) and/or work jointly to design and implement a program/project.” First, the informant needed to confirm that his or her organization had already been part of such an INGO–LNGO collaboration. Next, we asked each respondent to identify a collaboration with an INGO or LNGO that was complete or near completion so that its success could reasonably be assessed. We then requested that respondents answer survey questions based on this collaboration (Zacharia et al., 2011). For each NGO, we emailed the highest-ranked manager (e.g., head or director of mission or country, program director or project manager), for whom we obtained contact details from the OCHA, to participate in our study. We performed a series of prescreening checks to ensure the eligibility of the respondents. For example, a manager was deemed eligible to participate in our study if he or she was directly affiliated with the international (local) NGO and not affiliated with any United Nations (UN) agencies, Red Cross and Red Crescent, or a commercial organization. To ensure the competence of the respondents, we also captured the extent to which potential informants had been involved in collaboration activities and their knowledgeability regarding the organization, its services, and its collaboration-management practices and partners (Kumar et al., 1993). Specifically, we asked respondents to answer the following two questions using a 7-point Likert-type scale: (1) “I am knowledgeable about the collaboration or partnership activities between my organization and this partner” and (2) “I have been involved in the collaboration or partnership between my organization and this partner.” Respondents whose scale answers were 4 or below were excluded from further analysis.

Study 1 was carried out in March–April 2015, which resulted in 160 responses from INGOs and 140 responses from LNGOs. In our INGO sample, we removed 25 responses with missing data or unengaged respondents and filled out by informants lacking the desired competences, reducing the number of responses to 135 for a response rate of 17%. Regarding the LNGO sample, we followed the same procedure and removed 17 cases, reducing the total number of responses to 123 for a response rate of 13%. Table A1 in the appendix illustrates the Study 1 demographics. Taking late respondents as a proxy for nonrespondents, we tested for nonresponse bias using analysis of variance (ANOVA) on our dependent construct which yielded no significant difference between early-wave and late-wave groups of respondents, suggesting that nonresponse bias was not significantly present in our study.

### Measurement

We drew our multi-item 7-point Likert-type scales from the existing interdisciplinary literature, emphasizing collaboration, interorganizational relationships, NGOs, and humanitarian supply chains. To measure collaborative engagement, we reviewed and extracted the following scales from existing research (Zacharia et al., 2011): (CE1) joint goal setting for the collaboration effort, (CE2) joint decisions on most issues, (CE3) a free flow of useful ideas, and (CE4) open and two-way communication.

To measure the operational performance of the humanitarian project, we extracted five scales from prior studies (Zacharia et al., 2011) and adapted them to the humanitarian context (Balcik et al., 2010; Salem et al., 2019): (OP1) needs assessment accuracy, (OP2) delivery of products or services to beneficiaries, (OP3) costs, (OP4) quality of services to beneficiaries, and (OP5) safety or environmental performance. In addition to the satisfaction associated with the operational performance of collaboration efforts, we examined the responses of humanitarian partners regarding enhancement of local partners’ capabilities. To this end, we reviewed and extracted the scales from the interviews and practitioners’ reports (Villena & Craighead, 2017). These indicators are the local partner’s (LP1) organizational processes, (LP2) managerial skills, (LP3) engagement with local communities, and (LP4) engagement with the international humanitarian aid sector.

To measure the perceived stakeholder pressure to engage in collaboration, we used scales suggested by Sarkis et al. (2010). These scales assess the extent to which each organization feels pressure from the following stakeholders to engage in collaborations: (1) the government of the affected country, (2) donors, and (3) media. While single-item scales are not ideal, using them helps to avoid repetitive questions (Hoeppner et al., 2011), which we were encouraged to remove after the pilot phase, and shortens the overall survey length, which may increase response rates when participants have little time to fill out a questionnaire and limited internet connectivity (Salem et al., 2019).

In our analyses, we controlled for potential sources of heterogeneity. At the relationship level, we controlled for relationship duration, trust between partners and commitment as a component of relational orientation, and dependency perception. We also controlled for the scope of collaboration between partners, which refers to the range or types of tasks jointly carried out (Alcacer & Oxley, 2014). In a humanitarian setting, the scope of collaboration activities changes from the early planning stages to needs-assessment missions, context analysis, program design, and devising and preparing funding proposals. We operationalized the scope of collaboration at three levels and asked respondents to report on the extent to which the international (local) NGO has been involved in program implementation (e.g., distribution and delivery; CS1), in program design and development (e.g., needs assessments, context analysis, and resource mobilization; CS2), and in fundraising activities (e.g., proposal writing, contact with media, and contact with donors; CS3) in their local–international collaboration.

### Measurement Model Validity and Reliability

Prior to testing the proposed hypotheses, we followed several steps to validate our measures. First, we performed exploratory factor analysis on each multiple-item construct and confirmed that all items belonged to their prespecified constructs. In our international NGO sample, the values of Cronbach’s alpha for collaborative engagement, humanitarian project performance, and local partner capabilities constructs are .83, .83, and .76, respectively, suggesting the measures are internally consistent and reliable. Similarly, in the local NGO sample, the values of Cronbach’s alpha for collaborative engagement, humanitarian project performance, and local partner capabilities constructs are .90, .87, and .90, respectively, suggesting the measures are internally consistent and reliable. Tables A3 and A4 in the appendix provide descriptive statistics of our data.

We carried out a confirmatory factor analysis using Amos 28 to check the factor loadings, the constructs’ individual-item reliabilities, the convergent validity of the measures associated with each construct, and their discriminant validity. All items loaded highly on their latent factor (*p* < .01), ranging from 0.68 to 0.85 for the international sample and from 0.60 to 0.89 for the local (See Table A2 in the appendix). However, we removed an item from the humanitarian project performance constructs and one in local partner capabilities constructs in both samples because they performed poorly on the international sample (λ = 0.53 and 0.54). The results also suggest that the data fit our model reasonably well in both international (χ^2^_*d.f*.=62_ = 80, root mean square error of approximation [RMSEA] = 0.048, and CFI = 0.973) and local samples (χ^2^_*d.f*.=62_ = 105, RMSEA = 0.076, and CFI = 0.961). The composite reliabilities were all greater than 0.75, indicating good internal reliability. The average variance extracted of constructs meets the 0.50 threshold, suggesting convergent validity at the construct level. Overall, these statistics indicate that the psychometric properties of the model are sufficiently strong to enable the interpretation of structural estimates.

### Model Estimation and Analysis

Table 1 illustrates the results of standardized regression using the international NGOs sample. To test our hypotheses, we used a moderated regression analysis (Poppo et al., 2016). Considering the results of Model 1, we observe that some controls, such as collaboration scope and trust, are significant in explaining the variance of humanitarian project performance and local partner capabilities. Adding the independent variables to the regression in Model 2 significantly increased the *R*^2^ values to .48 and .48, respectively, and further including the interaction terms increased the *R*^2^ values by .08 (*p* < .05) and .04 (*p* < .01) in Model 3. To evaluate the explanatory power of the research model, we examined the explained variance (*R*^2^) of the endogenous constructs. The *R*^2^ values for project performance and local partner capabilities are moderate, at .56 and .52, respectively, in Model 3.

**Table 1.** Standardized Regression Results (INGOs, Study 1).

		Humanitarian project performance				Local partner capabilities		
Independent variables		Model 1	Model 2	Model 3		Model 1	Model 2	Model 3
Control variables								
Collaboration scope_I (implementation)		0.01	0.02	0.04		0.06	0.07	0.09
Collaboration scope_II (program design)		0.03	−0.01	−0.06		−0.22**	−0.26***	−0.28***
Collaboration scope_III (fundraising)		0.30***	0.34***	0.39***		0.29***	0.33***	0.35***
Trust		0.33***	0.30***	0.29***		0.19**	0.16*	0.16*
Commitment		0.06	0.04	0.06		0.28***	0.27***	0.28***
Relationship duration		0.08	0.08	0.06		0.01	0.01	−0.03
International NGO size		0.01	0.01	0.05		0.02	0.02	0.02
Local NGO size		−0.06	−0.06	−0.07		−0.14*	−0.15*	−0.15*
Dependency		0.20**	0.16*	0.16**		0.18**	0.15*	0.16*
Direct effects								
Collaborative engagement			0.28***	0.20***			0.26***	0.23***
Donor pressure			−0.09	−0.13			−0.07	−0.10
Government pressure			0.10	0.17			0.09	0.12
Media pressure			0.03	0.01			−0.05	−0.07
Moderating effects								
Collaborative engagement × DP	H1			−0.04	H2			0.08
Collaborative engagement × GP	H3			0.24***	H4			−0.02
Collaborative engagement × MP	H5			0.17**	H6			0.19**
*F* value		4.64***	4.75***	5.59***		4.76***	4.68***	4.66***
*R* ^2^		.42	.48	.56		.43	.48	.52
Adj *R*^2^		.33	.38	.46		.34	.38	.41
Δ *R*^2^			.06**	.08**			.05***	.04**

*Note.* INGO = international nongovernmental organizations; NGO = nongovernmental organizations; DP = donor pressure; GP = government pressure; MP = media pressure.

* *p* < .1. **p < .05. ***p < .01.

As for the main effects in Model 3, the signs of the estimated coefficients and their associated *p* values indicate that collaborative engagement has positive associations with both humanitarian project performance (*β* = 0.20. *p* < .01) and local partner capabilities (*β* = 0.23, *p* < .01; see Table 1, Model 3). In Model 3, we test our moderating variables. H1 and H2 proposed that the level of donor pressure positively moderates the relationship between collaborative engagement and our dependent variables. Surprisingly, our analysis revealed nonsignificant moderation effects of donor pressure on the link between collaborative engagement and both project performance and local partner capabilities. H3 and H4 predicted that government pressure moderates the relationship between collaborative engagement and collaboration outcomes. We found support for the moderating effect of government pressure on the association between collaborative engagement and humanitarian project performance (*β* = 0.24, *p* < .01), supporting H3. Finally, we found that media pressure positively moderates the link between collaborative engagement and both humanitarian project performance (*β* = 0.17, *p* < .05) and local partner capabilities (*β* = 0.19, *p* < .05), supporting both H5 and H6.

To better understand the potential differences in how LNGOs perceive the humanitarian project performance of their collaborative engagements, we repeated the entire analysis for the LNGO sample. Table 2 illustrates the results of standardized regression using the local NGOs sample. The results reveal that collaborative engagement in this sample also has positive associations with both humanitarian project performance (*β* = 0.21, *p* < .01) and local partner capabilities (*β* = 0.16, *p* < .01; see Table 2, Model 3). In our LNGO sample, donor pressure negatively moderates the path from collaborative engagement to local partner capabilities (*β* = −0.20. *p* < .05), whereas government pressure positively moderates the path from collaborative engagement to local partner capabilities (*β* = 0.18, *p* < .05), and media pressure positively moderates the path from collaborative engagement to humanitarian project performance (*β* = 0.19, *p* < .05).

**Table 2.** Standardized Regression Results (LNGOs, Study 1).

		Humanitarian project performance				Local partner capabilities		
Independent variables		Model 1	Model 2	Model 3		Model 1	Model 2	Model 3
Control variables								
Collaboration scope_I (implementation)		0.24**	0.27**	0.29**		0.25***	0.26***	0.35***
Collaboration scope_II (program design)		−0.09	−0.14	−0.18		0.24**	0.20*	0.17*
Collaboration scope_III (fundraising)		0.04	0.06	0.09		−0.12	−0.12	−0.16
Trust		0.43***	0.49***	0.55***		0.39***	0.43***	0.41***
Commitment		−0.03	−0.08	−0.09		0.04	0.03	0.08
Relationship duration		0.04	0.08	0.09		0.01	0.03	0.05
International NGO size		−0.10	−0.08	−0.10		0.01	0.01	−0.01
Local NGO size		−0.10	−0.10	−0.14		−0.04	−0.05	−0.09
Dependency		0.12	0.08	0.05		0.14	0.12	0.14
Direct effects								
Collaborative engagement			0.20**	0.21**			0.15**	0.16**
Donor pressure			0.07	0.04			0.03	0.02
Government pressure			−0.08	−0.07			−0.08	−0.12
Media pressure			0.20**	0.21**			0.07	0.13
Moderating effects								
Collaborative engagement × DP	H1			−0.02	H2			−0.20**
Collaborative engagement × GP	H3			0.08	H4			0.18**
Collaborative engagement × MP	H5			0.19**	H6			0.10
*F* value		4.51***	4.49***	4.32***		9.88***	8.49***	8.58***
*R* ^2^		.44	.50	.54		.63	.66	.70
Adj *R*^2^		.34	.39	.41		.57	.58	.62
Δ *R*^2^			.06**	.04**			.03**	.03**

*Note.* LNGO = local nongovernmental organizations; NGO = nongovernmental organizations; DP = donor pressure; GP = government pressure; MP = media pressure.

* *p* < .1. **p < .05. ***p < .01.

## Study 2

Since 2014, there have been more calls for aid localization and INGO–LNGO collaborations in humanitarian settings, and we expect to observe that stakeholder pressure will have a more powerful or even different relationship with collaborative engagement in later years (Charter for Change, 2016; Development Initiatives, 2016). We carried out a second study in 2020 to provide deeper insights into the introduction of the Sustainable Development Goals or the adoption of long-term funding by donors in the form of multi-year strategic response plans beginning in 2014 (Development Initiatives, 2016), which has manifested in the form of donor pressure, as well as to study the changing impact of other stakeholder pressures. We focused on the same population and followed an identical approach to the data collection of study 1 in contacting, inviting, and sending reminders to respondents from the same INGOs and LNGOs active in humanitarian activities. The data collection effort resulted in 197 responses from INGOs and LNGOs. We removed 40 cases with missing data, unengaged respondents, or filled out by ineligible informants, reducing the number of observations to 157 (80 from INGOs and 77 from LNGOs). Table A5 in the appendix illustrates the sample demographics in Study 2. Taking late respondents as a proxy for nonrespondents, we tested for nonresponse bias using ANOVA for each examined construct (Armstrong & Overton, 1977). The results for the international sample, that is, project performance (*F*[1, 48] = 0.003, *p* = .95) and local partner capabilities (*F*[1, 48] = 0.88, *p* = .35), and for the local sample, that is, project performance (*F*[1, 48] = 0.11, *p* = .73) and local partner capabilities (*F*[1, 48] = 0.35, *p* = .55), yielded no significant difference between early-wave and late-wave groups of respondents, suggesting that nonresponse bias is not significantly present in Study 2.

Before testing the same hypotheses with Study 2, we assessed the measurement model again. Tables A6 and A7 in the appendix provide detailed information about the validity and reliability of this measurement model. As in Study 1, our analyses indicate that our measures are valid and reliable.

To replicate our data analysis, we used moderated regression analysis to examine our hypotheses with the new study. As for Study 1, we used a residuals-based 3SLS approach to correct for endogeneity bias. Table 3 illustrates the estimation results. Collaborative engagement is positively associated with humanitarian project performance (*β* = 0.32, *p* < .01) from both INGOs’ and LNGOs’ perspectives. Moreover, we found that only in the INGO sample, donor pressure negatively (*β* = −0.28, *p* < .05) moderates the path from collaborative engagement to local partner capabilities, and there are significant positive moderation effects of government pressure on the path from collaborative engagement to both humanitarian project performance and local partner capabilities, except in the LNGO sample, where government pressure does not moderate the relationship between collaborative engagement and local partner capabilities. However, notably, media pressure negatively moderates the relationship between collaborative engagement and local partner capabilities in the LNGOs sample (*β* = −0.22, *p* < .05). This last finding is the opposite of what we found in Study 1.

**Table 3.** Standardized Regression Results (Study 2).

	INGOs		LNGOs	
Independent variables	Humanitarian project performance	Local partner capabilities	Humanitarian project performance	Local partner capabilities
Control variables				
Collaboration scope_I (implementation)	0.08	0.34**	0.06	0.14
Collaboration scope_II (program design)	−0.12	−0.32**	0.21*	0.18
Collaboration scope_III (fundraising)	0.02	0.13	−0.08	−0.05
Trust	0.17	0.11	−0.05	0.02
Commitment	−0.10	0.19	0.52***	0.55***
Relationship duration	0.05	0.30**	0.02	0.05
International NGO size	−0.14	0.01	−0.06	0.05
Local NGO size	0.19	0.08	−0.11	−0.05
Dependency	0.22*	0.35***	0.09	0.13
Mission	−0.03	−0.06	−0.12	0.06
Afghanistan	0.35**	0.10	0.21	0.05
Congo	0.54***	0.22	0.02	−0.03
Haiti	0.52***	0.04	0.03	−0.14
Indonesia	0.37**	0.16	0.15	−0.08
Kenya	0.41***	0.06	−0.05	−0.26**
Niger	0.43***	0.25	0.08	−0.16
Sudan	0.47***	0.39**	0.12	−0.03
Sri Lanka	0.16	0.09	−0.05	−0.08
Direct effects				
Collaborative engagement	0.32**	−0.10	0.32***	0.11
Donor pressure	0.11	0.25*	0.02	−0.18
Government pressure	−0.29	−0.26*	0.14	0.22**
Media pressure	0.05	−0.26*	0.08	0.17
Moderating effects				
Collaborative engagement × DP	−0.09	−0.28**	−0.12	0.06
Collaborative engagement × GP	0.33**	0.29**	0.23**	0.09
Collaborative engagement × MP	−0.08	0.07	−0.08	−0.22**
*F* value	2.36***	2.139***	4.11***	5.98***
*R* ^2^	.54	.51	.69	.56
Adj *R*^2^	.31	.27	.53	.44

*Note.* INGO = international nongovernmental organizations; LNGO = local nongovernmental organizations; NGO = nongovernmental organizations; DP = donor pressure; GP = government pressure; MP = media pressure.

* *p* < .1. **p < .05. ***p < .01.

## Discussion

In this article, we focused on the impact of stakeholder pressure on the NGO collaboration–performance relationship within the humanitarian context. Overall, this study confirms that collaborative engagement has positive associations with both humanitarian project performance and local partner capabilities. However, over time, we see that the relationship of collaborative engagement with local partner capabilities could not be confirmed. Across the two studies, our analyses also confirm the importance of external stakeholders in affecting the NGO collaboration–humanitarian performance relationship: Altogether, government pressure positively moderates the collaboration–performance link. However, our analyses indicate that initially INGOs believe that government pressure positively impacts the humanitarian project performance, while not local partner capabilities, whereas 5 years later, they also believe that this has an impact on local partner capabilities. LNGOs, however, show the opposite: They really seem to believe in the positive impact of government pressure on local partner capabilities, but this impact disappeared to just a positive impact on the humanitarian project performance. In contrast, donor pressure negatively affected the local partner capability developments as experienced by local NGOs and, in the longer term, also negatively impacted the local partner capability developments as experienced by the INGOs. Finally, media pressure changed the nature of its effects from positively to negatively moderating the association between NGO collaboration and humanitarian performance. While it originally shows the impact of collaborative engagement on performance, in later years, there seems to be a belief among LNGOs that media pressure weakens the impact of collaborative engagement on local partner capabilities. In the following sections, we discuss our findings and their relevant managerial implications in more detail.

### Collaborative Engagement and Humanitarian Performance

Consistent with previous research, Study 1 confirms that collaborative engagement is indeed positively associated with both humanitarian project performance and local partner capability enhancement. An interesting example in this respect is the Care–Live & Learn collaboration, in which Care International collaborates closely with Live & Learn, a local partner in Fiji, to undertake joint humanitarian projects. Although it began in Fiji, the collaboration has now expanded to other areas and has made important contributions to relief and development efforts (Lehoux, 2016). Indeed, both partners are convinced that this partnership not only created added value for both organizations but also—and more importantly—enabled the organizations to structurally meet the needs of cyclone-affected Fijians in an effective and sustainable manner. As such, these NGOs achieved together what neither could have done alone (Lehoux, 2016).

When the association between NGO collaboration and humanitarian performance is revisited in Study 2, our findings suggest that collaborative engagement is only linked to humanitarian project performance and not to local partner capabilities. One possible explanation for this may be that the effect of collaborative engagement on local partner capabilities is not sustainable enough in Study 2. This argument could be explained by paying attention to the role that INGOs predominantly assign to LNGOs—that is, implementers of international NGO programs but not really entities that become self-supporting. While both INGO and LNGO partners hoped that collaborative engagements would develop new capabilities in the field, this was apparently not in line with recent experiences. Indeed, collaborative engagement requires the investment of time and resources that goes beyond the capacity of LNGOs; that is, it necessitates greater engagement in collaborative activities like participation in meetings, planning, communication, and information sharing and reporting. These activities require time and resources and allow the organizations enough time to conduct their daily work in the field (i.e., actively helping people in emergencies and difficult situations). To decrease this administrative burden, NGO members of the ACT Alliance have formed a working group on harmonizing collaboration tools (Charter for Change 2019). The need to reduce this complexity has also been made explicit by LNGOs, which have stated that too many projects are not feasible, as they increase the complexity by increasing the number of reports, calendars, and formats for working with partners (De Geoffroy et al., 2017). Another plausible explanation may be the increasing demand for aid worldwide. Indeed, there has been a rise in the number of people in need over the last few years. The Development Initiatives (2020, p. 1) report reveals that “over one billion people were living in countries affected by long-term humanitarian crises such as conflict, displacement and natural disasters in 2019.” However, in the same period that these international UN appeals hit a record high, international humanitarian funding dropped by $1.6 billion. This surge in demand, in combination with less funding, may have resulted in rather short-term and smaller projects to deliver quick responses, instead of resource investments and capability-building projects for these local communities and NGOs.

### Stakeholder Pressure Matters

Study 1 shows that from LNGOs’ viewpoint, donor pressure has had a negative impact on the relationship between collaborative engagement and local partner capabilities, suggesting that high donor pressure attenuates the relationship between collaborative engagement and local partner capabilities. This finding is also supported by a recent survey of NGOs (OCHA, 2018), which revealed that 38% of NGOs had to appoint dedicated staff to comply with donor requirements, resulting in a diversion of resources that could have been used more effectively in the field to improve local capabilities. Donor requirements often create persistent blockages by limiting adequate consultation and co-creation with partners (Charter for Change 2019). In addition, tight spending timeframes to comply with donor requests create additional urgencies to respond, but they might just as easily undermine the efficiency of the humanitarian project (Nightingale, 2012). Because the risks of accountability and corruption are especially high in less-secured and more-remote areas, donors may require even higher levels of procedures and risk control mechanisms for these areas. These rationales behind donors’ tight requirements or policies are rather understandable for INGOs which are closer and more engaged with donors, so facing less psychological distance (Simpson et al., 2021). However, our results also indicate that this negative perceived impact moved from locals in Study 1 toward INGOs in Study 2. One explanation for this may be that donors shifted their focus to long-term performance implications. For example, in 2014, several donors introduced long-term funding projects based on multiyear strategic response plans (Development Initiatives, 2016). This long-term focus decreased the pressure on LNGOs because of the more stable financial support for these groups, but at the same time, it shifted the pressure to INGOs, which had to report more frequently and follow stricter guidelines to keep this long-term funding.

Government pressure brings more legitimacy and transparency to the collaborative engagements of NGOs; hence, this pressure reduces the operational risks and provides more local support and resources for the NGOs, which eventually may improve their humanitarian project performance. Governments create this legitimacy, for example, by receiving multiple types of operational permissions or requiring them to report regularly on the progress of collaborative projects. We noticed that these government pressures remained consistent in Studies 1 and 2. This indicates that governments might have an important role to play in supporting LNGOs and INGOs by steering them toward taking appropriate actions that increase the outcomes of humanitarian aid projects.

Nevertheless, in respect to the local partner capabilities, we noticed inconsistent results between Studies 1 and 2. Inconsistent with Study 1, INGOs in Study 2 perceive that governmental pressures could leverage local partner capabilities. This shift can be explained by the implementation of clearer rules and procedures by governments to ensure collaboration among NGOs (Stephens, 2016). Working within a stated range of activities (Mukhtar, 2020), not abandoning incomplete projects (Njeru, 2013), and transparency and accountability requirements (Anderson, 2017) are just a few of the rules that could enhance the capabilities of LNGOs. However, our data indicate that while LNGOs indeed expected these government rules and regulations to help increase their local capabilities, this perception changed toward impacting the humanitarian aid project but not the local capabilities per se. This might be explained by the increasing competition among LNGOs and higher pressure that LNGOs feel to provide short-term results, in line with the goals of the humanitarian project in recent years.

While the media cannot actively request the transparency of NGOs, they pressure INGOs by continuously keeping an eye on their operations. Media reports (e.g., reports on the progress in certain regions, specific projects, or on the use of budgets) can shape or change the reputation of an NGO, and as such, they could affect future donations the NGO receives. For example, in the summer of 2015, when the Danish media reported that the EU faced a large influx of refugees arriving on the Greek island of Lesbos, the Danish public criticized NGOs for their inactivity and pressured them to participate in the European territory and to make collaborations with LNGOs and volunteers succeed.

Inconsistent with the results in Study 1, our findings in Study 2 show nonsignificant and, in case of LNGOs, even a negative impact of media pressure on the collaboration–performance link. The surge in social media use, which disseminates information at an unprecedented pace and breadth, has placed time pressure on humanitarian operations (Moshtari et al., 2020). NGOs are now under huge media pressure, and thus, they are often forced to focus on rapid or even immediate tangible results (Bennett et al., 2006); this makes them less motivated or causes them to work on more nuanced efforts, such as improving local partner capabilities. In addition, NGOs are operating under increased stress levels because performance assessments and reports by the media are more widely and readily available than ever before. Likewise, in recent years, social media have played a crucial role as communication channels among beneficiaries, social media users, and humanitarian organizations. Given the challenges that beneficiaries face after a disaster, they increasingly communicate their expectations via social media to receive aid quickly. If this does not happen according to their expectations, they communicate the weak performance of humanitarian organizations widely via social media, which could put extra time pressure and stress on the humanitarian aid community to act, and therefore, sacrifice the long-term impact of their operations and developing local partners’ capabilities. Moreover, in recent years, there has been an increase in reporting negative news, such as the 120 aid workers who lost their jobs due to sexual misconduct in 2017 (Bacchi, 2017). When such negative information spreads, NGOs’ credibility and reputations among donors and governments could be threatened (Larché, 2011). In addition, negative information about what went wrong, such as cases of corruption, is more easily called to mind than what worked well (Kreidler, 2011).

### Limitations and Future Research

This research is not without limitations. While larger sample sizes would have allowed us to examine the research model in greater depth, it was already difficult to reach out to and persuade NGOs, especially LNGOs, to participate. While a response rate of 15% in Study 1 and 11% in Study 2 was certainly satisfactory, it could have been larger. Nevertheless, the small sample size was primarily due to the detailed inclusion criteria and the limited population eligible to participate. Testing the proposed hypotheses using other NGO groups that are not part of an OCHA cluster, or other humanitarian organizations beyond NGOs (e.g., World Food Programme or International Committee of the Red Cross), might provide additional insights, mainly in terms of stakeholder pressures and local development capabilities. However, broadening the scope also requires acknowledging differences in structures and overall goals of these humanitarian organizations, which may eventually also result in different perceptions of stakeholder pressures. Another avenue for future studies is to investigate the research constructs in detail. Another limitation of our research concerns its focus on ongoing collaborations. Future studies could focus on other stages of the relationship cycle by investigating, for instance, the influence of stakeholder pressure on the partner search and selection phases between INGOs and LNGOs. Finally, future studies may even use longitudinal or experimental methods to investigate the causality among the proposed relationships in this study.

### Managerial Implications

This study indicates that external stakeholders, such as donors, governments, and the media, play a crucial role in explaining the impact of collaborative engagement among partners on humanitarian performance. Based on our findings, we encourage all stakeholders to set policies or practices that create an environment in which collaboration among LNGOs and INGOs flourishes. Such policies should help NGOs effectively learn about one another’s goals, capabilities, and prior experiences and convince them to invest in relation-specific endeavors, such as interfaces and communication channels, knowledge-sharing routines, and dedicated human resources. These practices will facilitate collaboration among LNGOs and INGOs, ultimately making them more productive.

Donors need to consider balancing greater transparency in project implementation concerning the resources and time required to prepare documentation and concessions made to speedy and sometimes short-sighted projects at the expense of the long-term perspective. In this respect, we suggest that donors should follow a more capacity-strengthening approach instead of the project performance–based approaches that have become common in the humanitarian field. Such a capacity-strengthening approach requires the acceptance of donors to see national NGOs and LNGOs as direct eligible costs for providing humanitarian aid. On top of this, donors should also realize that working with national NGOs and LNGOs is not a risk-free endeavor because specific circumstances might affect its potential and success (De Geoffroy et al., 2017).

To further encourage the long-term orientation of local–international humanitarian NGO collaborations, donors and host governments can use both incentive mechanisms and penalty practices (e.g., providing or restricting access to the field, local information, and networks) to emphasize this long-term orientation of partnerships, as well as the long-term sustainability of LNGOs. Setting up well-balanced specific, measurable, assignable, realistic, and time-related (SMART) incentives, rules, and guidelines for local–international humanitarian NGO collaborations will enable these stakeholders to steer the NGOs into strengthening their benefits from collaboration. However, to set up these incentive mechanisms, it is important to further stimulate the debate between INGOs and LNGOs, including stakeholders. This may be done through the creation of forums or platforms to debate how to increase the impact of humanitarian aid projects. A recent example of an initiative bringing together international, national, and local actors is the creation of platforms and spaces for national and local actors to take on leadership roles. One notable outcome of such a platform, called reliefweb (see reliefweb.int), was an evidence-based examination of practices that were seen as most and least conductive to localization aid from both local and national humanitarian perspectives (Charter for Change, 2019). Other examples of such initiatives are the START network (https://startnetwork.org) or the CRS EMPOWER project (https://empower-project.eu/project). While these initiatives are new, further initiatives seeking to understand other parties’ perspectives, including and elaborating it toward stakeholders, might further boost the effectiveness of localizing humanitarian aid. Media could also play a crucial role in emphasizing the long-term, capability-strengthening objectives within local–international humanitarian NGO collaborations by focusing on sustainable solutions instead of rapid service delivery during humanitarian operations. This will require a completely different mentality from media that are keen on disseminating information in a timely fashion but with the drawback that the information is sometimes unverified and perhaps inaccurate. Focusing on more sustainable NGO outcomes will reduce the time pressure on NGOs and enable them to balance their attention in terms of multiple aspects of their humanitarian performance.

## Appendix

**Table A1.** Sample Demographics (Study 1).

	INGOs %	LNGOs %
Respondent’s position		
Head or director of mission or country	40	49
Head or director of program	39	27
Operations or logistics manager	12	5
Head of office	4	12
Other position	6	7
Respondent’s years of experience with organization		
0–2 years	23	14
2–5 years	45	30
More than 5 years	32	57
Organization’s number of employees		
X < 25	45	81
25 < X < 100	28	16
100 < X	27	2
Mission		
Disaster relief	28	13
Development aid	72	87
Organization’s type of service		
Agriculture	34	37
Health	44	50
Logistics	13	11
Emergency shelter	24	27
Nutrition	34	37
Protection	40	41
Water/sanitation	55	49
Education	45	59
Camp management/coordination	13	13
Early recovery	28	22
Other	14	11
Country		
Afghanistan	11	34
Congo	21	11
Haiti	21	11
Indonesia	10	6
Kenya	18	13
Niger	6	5
Sudan	7	5
Sri Lanka	6	14

*Note.* INGO = international nongovernmental organizations; LNGO = local nongovernmental organizations.

**Table A2.** Measurement Model I (Key Variables and Respective Items, Study 1).

Latent variables and respective items	Factor loading	
	INGOs	LNGOs
Humanitarian project/program performance (Nyaga et al., 2010; Zacharia et al., 2011) (INGO: CR = 0.85, AVE = 0.58) (LNGO: CR = 0.88, AVE = 0.59) Collaboration between international and local organizations has . . .		
increased our needs assessment accuracy (OP1)	0.73	0.86
resulted in quicker delivery of products or services (OP2)	0.83	0.77
resulted in lower costs (OP3)	0.53^a^	0.60
resulted in better services to beneficiaries (OP4)	0.81	0.80
resulted in a better safety, environmental, or regulatory performance (OP5)	0.68	0.80
Local partner capabilities (Villena & Craighead, 2017) (INGO: CR = 0.76, AVE = 0.52) (LNGO: CR = 0.91, AVE = 0.70) Collaboration between international and local organizations . . .		
improved local partner’s organizational processes (LP1)	0.75	0.84
improved local partner’s managerial skills (LP2)	0.72	0.87
improved local partner’s engagement with local communities (LP3)	0.54^a^	0.80
improved local partner’s engagement with international humanitarian aid sector (LP4)	0.69	0.84
Collaborative engagement (Zacharia et al., 2011) (INGO: CR = 0.83, AVE = 0.56) (LNGO: CR = 0.90, AVE = 0.69)		
The organizations involved jointly set goals for the collaboration effort (CE1)	0.68	0.77
The organizations involved made joint decisions on most issues (CE2)	0.68	0.78
Throughout this collaboration, there was a free flow of useful ideas (CE3)	0.77	0.89
Both organizations had open, two-way communication (CE4)	0.85	0.87
Government pressure (Sarkis et al., 2010)		
To what extent does your organization feel pressure from the government of the affected country to engage in collaborative relationships? (GP)	^b^	^b^
Donor pressure (Sarkis et al., 2010)		
To what extent does your organization feel pressure from donors to engage in collaborative relationships? (DP)	^b^	^b^
Media pressure (Sarkis et al., 2010)		
To what extent does your organization feel pressure from the media to engage in collaborative relationships? (MP)	^b^	^b^

*Note.* We modified these items in the online survey so that INGOs and LNGOs would see only “their” version. All measures used a 7-point Likert-type scale (1 = “*strongly disagree*” to 7 = “*strongly agree*”). INGO = international nongovernmental organizations; LNGO = local nongovernmental organizations; AVE = average variance extracted; LP = local partner capabilities; CE = collaborative engagement; GP = government pressure; DP = donor pressure; MP = media pressure.

a Item dropped because of poor psychometric properties. ^b^ Factor loading cannot be computed because this is a single-item factor.

**Table A3.** Means, Standard Deviations (SD), Intercorrelations, Fornell–Larcker, and HTMT Criterion Results (Study 1, INGO Sample).

variable	*M*	*SD*	CE	HPP	LPC	GP	DP	MP	CS1	CS2	CS3	TR	CO	RD	LNS	INS	DE	MI
CE	5.54	0.91	(.75)	.540^a^	.690^a^	—	—	—	—	—	—	—	—	—	—	—	—	—
HPP	5.23	1.11	.481**	(.76)	.635^a^	—	—	—	—	—	—	—	—	—	—	—	—	—
LPC	5.55	0.90	.546**	.550**	(.72)	—	—	—	—	—	—	—	—	—	—	—	—	—
GP	2.84	1.2	−.01	.05	.08	—	—	—	—	—	—	—	—	—	—	—	—	—
DP	3.31	1.12	−.04	.08	.02	.468**	—	—	—	—	—	—	—	—	—	—	—	—
MP	2.19	1.05	−.10	0.15	.09	.450**	.500**	—	—	—	—	—	—	—	—	—	—	—
CS1	6.11	0.90	.358**	.170*	.212*	−.02	.00	−.01	—	—	—	—	—	—	—	—	—	—
CS2	5.5	1.34	.358**	.339**	.15	.02	.07	.06	.452**	—	—	—	—	—	—	—	—	—
CS3	4.45	1.69	.337**	.371**	.253**	−.07	.01	.10	.196*	.561**	—	—	—	—	—	—	—	—
TR	5.56	1.03	.613**	.466**	.425**	.06	.13	.13	.182*	.291**	.15	—	—	—	—	—	—	—
CO	5.56	1.14	.457**	.286**	.374**	.02	.10	.11	.257**	.220*	.10	.558**	—	—	—	—	—	—
RD	2.23	0.77	−.05	.16	.13	.04	−.01	.01	−.11	.10	.08	.03	.05	—	—	—	—	—
LNS	1.81	0.81	.02	−.04	−.199*	−.15	−.08	−.04	−.07	.05	.13	−.06	.01	−.08	—	—	—	—
INS	1.87	0.84	−.10	.00	.02	.10	.06	.06	−.13	.00	−.02	−.03	.06	.01	−.08	—	—	—
DE	5.27	1.43	.15	.279**	.178*	.05	.11	.08	.08	.14	.02	.219*	.16	.181*	.12	.13	—	—
MI	1.73	0.45	−.03	−.13	−.08	.03	.07	−.05	.04	.05	−.03	.01	.00	.12	.08	−.04	−.02	—

*Note.* Fornell–Larcker criterion results (square roots of AVEs) are provided in parentheses for the three multi-item constructs. INGO = international nongovernmental organization; CE = collaborative engagement; HPP = humanitarian project performance; LPC = local partner capabilities; GP = government pressure; DP = donor pressure; MP = media pressure; CS = collaboration scope; TR = trust; CO = commitment; RD = relationship duration; LNS = local NGO size; INS = international NGO size; DE = dependency; MI = mission; AVE = average variance extracted.

a The HTMT criterion (where applicable) is shown in parentheses.

* Correlation is significant at the .05 level (2-tailed). **Correlation is significant at the .01 level (2-tailed).

**Table A4.** Means, Standard Deviations (SD), Intercorrelations, Fornell–Larcker, and HTMT Criterion Results (Study 1, LNGO Sample).

	*M*	*SD*	CE	HPP	LPC	GP	DP	MP	CS1	CS2	CS3	TR	CO	RD	LNS	INS	DE	MI
CE	5.56	1.13	(.83)	.650^a^	.798^a^	—	—	—	—	—	—	—	—	—	—	—	—	—
HPP	5.43	1.16	.583**	(.77)	.793^a^	—	—	—	—	—	—	—	—	—	—	—	—	—
LPC	5.72	1.20	.718**	.698**	(.84)	—	—	—	—	—	—	—	—	—	—	—	—	—
GP	2.92	1.32	.054	.074	−.033	—	—	—	—	—	—	—	—	—	—	—	—	—
DP	2.95	1.30	−.121	−.022	−.083	.437**	—	—	—	—	—	—	—	—	—	—	—	—
MP	2.41	1.14	.109	.145	.045	.505**	.422**	—	—	—	—	—	—	—	—	—	—	—
CS1	5.99	1.20	.644**	.497**	.619**	−.099	−.157	−.057	—	—	—	—	—	—	—	—	—	—
CS2	5.54	1.49	.650**	.453**	.622**	−.061	−.109	−.016	.683**	—	—	—	—	—	—	—	—	—
CS3	5.25	1.54	.555**	.449**	.514**	−.010	−.096	−.111	.653**	.742**	—	—	—	—	—	—	—	—
TR	5.70	1.35	.718**	.630**	.717**	.041	−.171	−.030	.556**	.601**	.546**	—	—	—	—	—	—	—
CO	5.62	1.32	.644**	.463**	.569**	.020	.008	.106	.523**	.588**	.485**	.695**	—	—	—	—	—	—
RD	2.36	0.75	.139	.211*	.252**	−.018	−.017	−.117	.114	.249**	.298**	.319**	.184*	—	—	—	—	—
LNS	1.96	0.80	−.072	.036	.015	−.003	−.076	−.045	.163	.035	−.036	.057	.080	−.032	—	—	—	—
INS	1.87	0.78	−.076	−.150	−.076	−.042	−.039	−.142	.082	−.016	.077	−.075	.010	.176	.253*	—	—	—
DE	5.53	1.52	.522**	.476**	.528**	.149	.030	.057	.397**	.494**	.478**	.581**	.502**	.316**	−.017	−.100	—	—
MI	1.87	0.34	.149	.076	.040	.050	.153	.078	−.063	.042	.001	.075	.053	0.141	−.080	.028	−.041	—

*Note.* Fornell–Larcker criterion results (square roots of AVEs) are provided in parentheses for the three multi-item constructs. CE = collaborative engagement; HPP = humanitarian project performance; LPC = local partner capabilities; GP = government pressure; DP = donor pressure; MP = media pressure; CS = collaboration scope; TR = trust; CO = commitment; RD = relationship duration; LNS = local NGO size; INS = international NGO size; DE = dependency; MI = mission; AVE = average variance extracted.

a The HTMT criterion (where applicable) is shown in parentheses.

* Correlation is significant at the .05 level (2-tailed). **Correlation is significant at the .01 level (2-tailed).

**Table A5.** Sample Demographics (Study 2).

Respondent’s position	INGOs %	LNGOs %
Head or director of mission or country	38	37
Head or director of program	37	42
Operations or logistics manager	5	3
Head of office	16	10
Other positions	4	8
0–2 years	25	13
2–5 years	29	29
More than 5 years	45	58
Organization’s number of employees		
X < 25	48	34
25 < X < 100	30	51
100 < X	22	15
Mission		
Disaster relief	32	10
Development aid	68	90
Organization’s type of service		
Nutrition	51	38
Health	36	47
Water/Sanitation	6	5
Camp management/coordination	18	14
Protection	30	38
Early recovery	44	62
Logistics	49	49
Agriculture	48	58
Emergency shelter	4	0
Education	4	8
Emergency telecommunication	24	17
Others	16	28
Country		
Afghanistan	3	16
Congo	18	33
Haiti	15	13
Indonesia	7	4
Kenya	5	8
Niger	15	12
Sudan	3	3
Sri Lanka	34	11

*Note.* INGO = international nongovernmental organizations; LNGO = local nongovernmental organizations.

**Table A6.** Means, Standard Deviations (SD), and Intercorrelations (Study 2, INGO Sample).

	*M*	*SD*	CE	HPP	LPC	GP	DP	MP	CS1	CS2	CS3	TR	CO	RD	LNS	INS	DE	MI
CE	5.67	0.99	—	—	—	—	—	—	—	—	—	—	—	—	—	—	—	—
HPP	5.44	0.91	.442**	—	—	—	—	—	—	—	—	—	—	—	—	—	—	—
LPC	5.86	0.75	.298**	.581**	—	—	—	—	—	—	—	—	—	—	—	—	—	—
GP	2.93	1.34	−.07	−.241*	−.21	—	—	—	—	—	—	—	—	—	—	—	—	—
DP	3.11	1.26	−.02	−.04	−.09	.567**	—	—	—	—	—	—	—	—	—	—	—	—
MP	2.23	1.01	−.18	.07	−.21	.380**	.378**	—	—	—	—	—	—	—	—	—	—	—
CS1	6.24	1.08	.524**	.337**	.344**	.03	−.10	−.16	—	—	—	—	—	—	—	—	—	—
CS2	5.64	1.32	.445**	.287*	.230*	−.02	.01	−.07	.609**	—	—	—	—	—	—	—	—	—
CS3	4.81	1.72	.302**	.268*	.359**	−.09	−.03	−.05	.391**	.386**	—	—	—	—	—	—	—	—
TR	5.56	1.34	.475**	.434**	.346**	−.10	.04	.02	.413**	.523**	.348**	—	—	—	—	—	—	—
CO	5.66	1.41	.541**	.325**	.307**	−.14	−.15	−.17	.394**	.340**	.279*	.691**	—	—	—	—	—	—
RD	2.46	0.66	.01	.10	.16	−.06	.04	.07	−.11	.02	−.01	−.03	−.10	—	—	—	—	—
LNS	1.35	0.56	.21	.233*	.13	.04	−.08	.08	.07	.227^*^	−.05	.17	.21	.06	—	—	—	—
INS	1.74	0.81	.233*	−.08	.01	.09	.00	−.15	.07	.09	−.227*	.06	.00	−.05	.313**	—	—	—
DE	5.2	1.59	.16	.278*	.302**	−.11	−.09	.06	.289**	.252*	.338**	.220*	.07	−.03	−.02	−.11	—	—
MI	1.69	0.47	.07	−.01	.06	−.18	.08	.05	−.08	.02	−.03	.02	−.05	.13	−.03	−.19	.09	—

*Note.* INGO = international nongovernmental organizations; CE = collaborative engagement; HPP = humanitarian project performance; LPC = local partner capabilities; GP = government pressure; DP = donor pressure; MP = media pressure; CS = collaboration scope; TR = trust; CO = commitment; RD = relationship duration; LNS = local NGO size; INS = international NGO size; DE = dependency; MI = mission.

** Correlation is significant at the .01 level (2-tailed). *Correlation is significant at the .05 level (2-tailed).

**Table A7.** Means, Standard Deviations (SD), and Intercorrelations (Study 2, LNGO Sample).

	*M*	*SD*	CE	HPP	LPC	GP	DP	MP	CS1	CS2	CS3	TR	CO	RD	LNS	INS	DE	MI
CE	5.30	1.33	—	—	—	—	—	—	—	—	—	—	—	—	—	—	—	—
HPP	5.23	1.31	.717**	—	—	—	—	—	—	—	—	—	—	—	—	—	—	—
LPC	5.62	1.26	.607**	.671**	—	—	—	—	—	—	—	—	—	—	—	—	—	—
GP	2.94	1.24	.066	.179	.150	—	—	—	—	—	—	—	—	—	—	—	—	—
DP	2.96	1.28	.031	.098	.173	.504**	—	—	—	—	—	—	—	—	—	—	—	—
MP	2.42	1.26	.086	.117	.232*	.384**	.509**	—	—	—	—	—	—	—	—	—	—	—
CS1	5.74	1.50	.475**	.484**	.525**	−.030	.056	−.132	—	—	—	—	—	—	—	—	—	—
CS2	5.40	1.66	.557**	.367**	.410**	−.025	−.023	−.078	.521**	—	—	—	—	—	—	—	—	—
CS3	4.67	1.86	.343**	.309**	.208	.002	.027	−.159	.332**	.672**	—	—	—	—	—	—	—	—
TR	5.59	1.12	.609**	.539**	.675**	.050	.048	.061	.433**	.371**	.191	—	—	—	—	—	—	—
CO	5.39	1.32	.623**	.631**	.614**	.047	.017	−.055	.470**	.346**	.332**	.742**	—	—	—	—	—	—
RD	2.53	0.70	.106	.140	.366**	−.035	−.027	.152	.196	.057	−.016	.298**	0.225	—	—	—	—	—
LNS	1.66	0.76	.162	.004	.071	.010	−.070	−.162	.186	.065	−.099	.144	0.160	.181	—	—	—	—
INS	1.78	0.66	−.012	−.065	−.003	−.129	−.164	−.299**	.180	.092	.226*	−.106	−0.007	.030	.254*	—	—	—
DE	5.15	1.72	.440**	.492**	.519**	.163	.020	.251*	.393**	.314**	.142	.363**	.417**	.236*	.094	.064	—	—
MI	1.90	0.31	−.042	−.135	.050	−.086	−.043	.079	−.087	.133	.213	−.076	−0.080	.200	−.044	.081	−.217	—

*Note.* LNGO = local nongovernmental organizations; CE = collaborative engagement; HPP = humanitarian project performance; LPC = local partner capabilities; GP = government pressure; DP = donor pressure; MP = media pressure; CS = collaboration scope; TR = trust; CO = commitment; RD = relationship duration; LNS = local NGO size; INS = international NGO size; DE = dependency; MI = mission.

* Correlation is significant at the .05 level (2-tailed). **Correlation is significant at the .01 level (2-tailed).
